# Inhibition of Phosphatase Activity Follows Decline in Sulfatase Activity and Leads to Transcriptional Effects through Sustained Phosphorylation of Transcription Factor MITF

**DOI:** 10.1371/journal.pone.0153463

**Published:** 2016-04-14

**Authors:** Sumit Bhattacharyya, Leo Feferman, Joanne K. Tobacman

**Affiliations:** 1 Department of Medicine, University of Illinois at Chicago, Chicago, Illinois, 60612, United States of America; 2 Jesse Brown VA Medical Center, Chicago, Illinois, 60612, United States of America; University of Padova, ITALY

## Abstract

Arylsulfatase B (B-acetylgalactosamine 4-sulfatase; ARSB) is the enzyme that removes 4-sulfate groups from the non-reducing end of the glycosaminoglycans chondroitin 4-sulfate and dermatan sulfate. Decline in ARSB has been shown in malignant prostate, colonic, and mammary cells and tissues, and decline in ARSB leads to transcriptional events mediated by galectin-3 with AP-1 and Sp1. Increased mRNA expression of GPNMB (transmembrane glycoprotein NMB) in HepG2 cells and in hepatic tissue from ARSB-deficient mice followed decline in expression of ARSB and was mediated by the microphthalmia-associated transcription factor (MITF), but was unaffected by silencing galectin-3. Since GPNMB is increased in multiple malignancies, studies were performed to determine how decline in ARSB increased GPNMB expression. The mechanism by which decline in ARSB increased nuclear phospho-MITF was due to reduced activity of SHP2, a protein tyrosine phosphatase with Src homology (SH2) domains that regulates multiple cellular processes. SHP2 activity declined due to increased binding with chondroitin 4-sulfate when ARSB was reduced. When SHP2 activity was inhibited, phosphorylations of p38 mitogen-associated phosphokinase (MAPK) and of MITF increased, leading to GPNMB promoter activation. A dominant negative SHP2 construct, the SHP2 inhibitor PHSP1, and silencing of ARSB increased phospho-p38, nuclear MITF, and GPNMB. In contrast, constitutively active SHP2 and overexpression of ARSB inhibited GPNMB expression. The interaction between chondroitin 4-sulfate and SHP2 is a novel intersection between sulfation and phosphorylation, by which decline in ARSB and increased chondroitin 4-sulfation can inhibit SHP2, thereby regulating downstream tyrosine phosphorylations by sustained phosphorylations with associated activation of signaling and transcriptional events.

## Introduction

The present study was undertaken to determine the transcriptional mechanism by which decline in ARSB increased GPNMB (transmembrane glycoprotein NMB; glycoprotein non-metastatic melanoma protein B; osteoactivin) expression in hepatocytes. GPNMB was identified on a cDNA microarray of ARSB-null mouse hepatic tissue as the only gene that was significantly upregulated [1]. GPNMB has been shown to be increased in multiple malignancies, including breast cancer, prostate cancer, glioblastoma multiforme, melanoma, gastric cancer, colorectal carcinoma, small cell lung cancer, renal cell carcinoma, and hepatocellular carcinoma [2–11]. Increases have been associated with propensity for metastases, and anti-GPNMB directed monoclonal antibody therapy has been therapeutically beneficial [12–14].

In this report, we present a transcriptional mechanism by which decline in ARSB and increase in chondroitin 4-sulfate (C4S) lead to increased expression of GPNMB through the microphthalmia-associated transcription factor (MITF)-binding site in the GPNMB promoter. MITF has been identified as the transcription factor required for promoter activation and increased GPNMB expression in melanoblasts, osteoblasts, and dendritic cells [15–17]. In previously published work, galectin-3 was shown to mediate transcriptional effects of ARSB and chondroitin 4-sulfate [18–20], and decline in ARSB had been associated with mammary, colonic, and prostatic malignancies [21–25]. Initial experiments indicated that silencing galectin-3 did not affect the expression of GPNMB, and further investigation addressed elucidation of an alternative transcriptional mechanism. This mechanism, as detailed in this report, involves increased binding of the tyrosine phosphatase SHP2 to the more highly sulfated C4S present when ARSB is reduced, in contrast to the reduced binding of galectin-3 to more highly sulfated C4S when ARSB is reduced.

The signaling pathways stimulated by decline in ARSB involve chondroitin 4-sulfate (C4S), dermatan sulfate (DS), or sulfate, since the only known biochemical function of ARSB is to remove 4-sulfate groups from the N-acetylgalactosamine 4-sulfate residue at the non-reducing end of C4S or DS. The transcriptional effects of ARSB and C4S due to reduced binding of galectin-3 to the more highly sulfated chondroitin 4-sulfate present when ARSB was reduced increased expression of versican in prostate cells, Wnt9A in colonic epithelial cells, and HIF-1α in bronchial epithelial cells [18–20]. Nuclear galectin-3 increased and interacted with Activator Protein (AP)1 and Specificity Protein (Sp)1 to enhance promoter activation and gene expression.

The current study findings present a novel integration between sulfation and phosphorylation, due to the effect of ARSB on the regulation of the SHP2 phosphatase. SHP2 is a ubiquitous, intracellular, SH2-containing protein tyrosine phosphatase that is encoded by PTPN11, and reduced SHP2 activity leads to increase in the downstream phosphorylation of p38-MAPK [26–31]. In this report, additional downstream effects of SHP2 inhibition on phospho-MITF and on GPNMB promoter activation are shown. The experiments in this report support a mechanism by which sulfation can regulate some phosphorylations, through the inhibition of the SHP2 phosphatase action. SHP2 is required for removal of tyrosine phosphates of SH2 domains, thereby regulating a broad range of important signaling events, including those dependent on p38 MAPK tyrosine phosphorylation. Since p38 tyrosine phosphorylation can trigger Ser-Thr phosphorylations [29–31], the inhibition of SHP2 by increased chondroitin 4-sulfation can exert a profound impact on other downstream phosphorylations and signaling, involving Ser-Thr phosphorylations.

Although previously considered as only an intracellular lysosomal enzyme associated with the inherited metabolic deficiency disorder Mucopolysaccharidosis VI, other reports with imaging have identified ARSB on the cell membrane of epithelial and endothelial mammalian cells, leading to characterization of ARSB as not just a lysosomal enzyme [32–34]. Other findings have indicated that acquired deficiency of ARSB may arise due to hypoxia or chloride excess [20,33], providing an opportunity for integration between environmental conditions and ARSB-mediated effects. ARSB is well-positioned to remove sulfate groups from C4S on the cell surface or extracellular matrix, as well as from C4S present at intracellular sites.

## Materials and Methods

### Cell culture and inhibitor and inhibitors

The HepG2 cell line (HB-8065) was obtained (ATCC, Manassas, VA) and grown in minimum essential medium (MEM) with 10% FBS at 37°C in a humidified, 5% CO_2_ environment, with exchange of media every 2–3 days. Confluent cells in T-25 flasks were harvested by EDTA-trypsin, and sub-cultured in multiwell tissue culture plates under similar conditions. Cells were treated by siRNA, DNA vectors for overexpression, and control vectors, as described below, and by enzyme and peptide inhibitors. Inhibitors included: genistein, a tyrosine kinase inhibitor (Sigma-Aldrich Co, St. Louis, MO; 10μM); SB203580, a p38 MAPK inhibitor (EMD Millipore, Billerica, MA; 10μM); LY294002 [2-(4-morpholinyl)-8-phenyl-1(4*H*)-benzopyran-4-one hydrochloride; EMD Millipore; 50μM), a phosphoinositide 3-kinase (PI3K)/AKT inhibitor, and PHSP1, a chemical SHP2 inhibitor (phenylhydrazonopyrazolone sulfonate; Sigma-Aldrich; 30μM) [35]. These inhibitors were dissolved in media, and HepG2 cells were exposed for 24 h.

### ARSB-deficient mouse model

Eight-week old, heterozygous, arylsulfatase B-deficient mice were obtained (Jackson Laboratories, Bar Harbor, Maine; Strain 005598) and housed in the Biological Resources Laboratory (BRL) of the University of Illinois at Chicago (UIC) or the Veterinary Medicine Unit at the Jesse Brown Veterans Affairs Medical Center (JBVAMC; Chicago, IL). The Institutional Animal Care and Use Committees of UIC and JBVAMC approved this research. The animals were bred as detailed previously to make a colony of homozygous arylsulfatase B-null mice [18]. Animals were maintained with routine light-dark cycles and standard mouse chow and drank water *ad libitum*. Weights were measured routinely, and the animals were euthanized by carbon dioxide inhalation and cervical dislocation. Hepatic tissue was harvested promptly and frozen at -80°C pending experiments.

### cDNA microarray of ARSB-null and control mouse hepatic tissue

cDNA microarray was performed using ARSB-null hepatic tissue and C57BL/6J control hepatic tissue with the Affymetrix GeneChip MoGene-2_-st-v1 mouse array [1]. Samples were labeled and hybridized according to the recommended labeling protocol, and array images were analyzed for quality metrics. Data were analyzed using Partek Genomics statistical package, and signal intensities were normalized by quantiles and summarized. ANOVA tests were used to calculate the significance of the differential gene expression of GPNMB, and other genes, between null and control mouse samples.

### Measurement of ARSB and N-acetylgalactosamine 6-sulfatase (GALNS) activity

ARSB activity in the control and treated cells was determined as reported previously [21,22]. Briefly, tissue or cells were harvested, homogenates were prepared, and ARSB activity in the various samples was determined using 4-methylumbelliferyl sulfate (MUS) as substrate in 0.05M acetate buffer, at pH 5.6. ARSB activity was expressed as nmol / mg protein / hour. GALNS activity was measured as previously reported [21,22], using exogenous substrates (from Moscerdam Substrates, Rotterdam, the Netherlands and Sigma-Aldrich). Five μl of cell homogenate in ddH_2_O treated by sonication with metal tip was combined with 5 μl 0.2% heat-inactivated BSA and 20 μl of the substrate [10 mM 4-methylumbilliferyl-ß-D-galactoside-6-sulfateNH_4_ (MU-ßGal-6S)] in substrate buffer made of 0.1M sodium acetate / 0.1M acetic acid at pH 4.3 with 0.1M NaCl, 5mM Pb-acetate (1.9 mg/ml) and 0.02% Na-azide. A microtiter plate was prepared, sealed, and incubated for 17 hours at 37˚C, and then 5 μl 0.9 M Na-Phosphate buffer at pH 4.3 with 0.02% Na-azide and 10 μl of 10U ß-D-galactoside galactohydrolase (Sigma) / ml 0.2% heat-inactivated BSA were added. The plate was incubated for 2 h at 37˚C, and then 200 μl of stop buffer (0.5M NaHCO_3_ / 0.5M Na_2_CO_3_ at pH 10.7 with 0.025% Triton-X-100) was added, and readings were taken at 360 nm and 465 nm. GALNS activity is expressed as nmol/hr/mg protein.

### mRNA expression of lysosomal enzymes

Primers were selected to detect the mRNA expression of lysosomal enzymes including ARSB, GALNS, α-galactosidase, and TFEB in the HepG2 cells following ARSB knockdown [36]. QRT-PCR was performed as previously described [23]. Primers were: ARSB (NM_000046) (left) 5´-AGA CTT TGG CAG GGG GTA AT- 3´ and (right) 5´-CAG CCA GTC AGA GAT GTG GA-3´; GALNS (NM_000512) (left) 5´- ACG GAT TTG ATG AGT GGT TTG -3´and (right) 5´- GTA GAG GAA AAA GGG GTG GTG—3´; α-Galactosidase A (X05790) (left) 5´- TGG AAG GAT GCA GGT TAT GAG—3´ and (right) 5´- CCC TAG CTT CAG TCC TTT GCT- 3´; and LFEB (NM_007162.2) (left) - 5´-TGA TCC ACT TCT GTC CAC CA-3´ and (right) 5´-CAG GTG GCT ACT TCA CAC ACA-3´.

### ARSB and Galectin-3 silencing by siRNA and ARSB overexpression

Specific siRNAs for ARSB and galectin-3 and control siRNAs were procured (Qiagen, Valencia, CA). Effectiveness of silencing was demonstrated by measurements of ARSB protein, mRNA, and activity and galectin-3 protein [18,19,22]. Cells were grown to 60–70% confluency in 12-well tissue culture clusters, and the medium of the growing cells was aspirated and replaced with 1.1 ml of fresh medium with serum. 0.3 μl of 20μM siRNA (75 ng) was mixed with 100 μl of serum-free medium and 6 μl of HiPerfect Transfection Reagent (Qiagen), as previously [18,19,22]. The mixture was incubated at room temperature for 10 minutes to allow the formation of transfection complexes, and then added dropwise onto the cells. The plates were swirled gently, and treated cells were incubated at 37°C in a humidified 5% CO_2_ environment. After 24 hours, the medium was exchanged with fresh growth medium. Effectiveness of silencing was determined by measurement of ARSB activity or specific quantification by ELISA assay (MyBioSource, San Diego, CA).

### Transfections of ARSB and SHP2

ARSB (NM_000046) plasmid in pCMV6-XL4 vector was obtained (Origene), and overexpressed in HepG2 cells by transient transfection using 2 μg of the plasmid and Lipofectamine™ 2000 (Invitrogen) [20,22]. Controls included untransfected cells and cells transfected with ARSB vector control. Media were changed after 6 h, and cells were incubated in humidified, 37°C, 5% CO_2_ environment and then harvested 24 h after transfection. ARSB activity was determined following transfection, as previously [21,22]. SHP2 dominant negative (DN), constitutively active (CA), wild type (WT), and empty vector plasmids were obtained (from Dr. Stuart Frank, University of Alabama at Birmingham) [37] and transfected into the HepG2 cells by Lipofectamine™ 2000. Efficiency of transfection was determined by measurements of SHP2 protein or SHP2 activity by ELISA (R&D, Minneapolis, MN).

### Measurement of total sulfated glycosaminoglycans

Total sulfated glycosaminoglycans (GAGs) in the cell lysates and tissues were measured using the sulfated GAG assay (Blyscan^TM^, Biocolor Ltd., Newtownabbey, Northern Ireland) [22]. The sulfated polysaccharide component of proteoglycans and protein-free sulfated GAG chains were measured, whereas hyaluronan and degraded disaccharide fragments were not detected. The reaction was performed in the presence of excess unbound 1,9-dimethylmethylene blue dye. The cationic dye and sulfated GAG at acid pH produced an insoluble dye-GAG complex, and the sulfated GAG content was determined by the amount of dye that was recovered from the test sample following exposure to Blyscan dissociation reagent. Absorbance maximum of 1,9-dimethylmethylene blue was at 656 nm, and sulfated GAG concentration was expressed as μg / mg of protein of cell or tissue lysate.

### Measurement of chondroitin 4-sulfate

Chondroitin 4-sulfate antibody (C4S; Ly111; Seikagaku, Amsbio, Lake Forest, CA) was used to immunoprecipitate C4S from cell lysates of treated and control cells [22]. The precipitate was eluted with dye-free elution buffer and subjected to sulfated GAG assay, as above.

### HepG2 cell survival following ARSB silencing and exposure to enzyme inhibitors

Proportions of live and dead HepG2 cells were detected using calcein-AM and ethidium homodimer-1 (EthD-1) in the live-dead cell assay with established procedures (Molecular Probes, Eugene, OR) [38]. HepG2 cells were grown for 24 h, then treated with the inhibitors genistein 10μM; SB203580 10μM; and LY294002 50μM for 24 h and harvested following incubation with calcein-AM and EthD-1 for 45 min. The percentage of live cells was detected by calcein-AM uptake and metabolism, and the percentage of dead cells was detected by EthD-1 uptake, measured by fluorescence with readings at 485/530 for calcein-AM and at 530/645 for EthD-1.

### Western blot for nuclear phospho-MITF

Nuclear extracts were prepared by using a nuclear extraction kit (Active Motif, Carlsbad, CA) following exposure to control or ARSB siRNA. Anti-phospho(Ser180/73)-MITF rabbit polyclonal antibody (Sigma-Aldrich) was used to detect nuclear phospho-MITF by Western blot. Lamin-A (Calbiochem, EMD Millipore) was used as a nuclear protein control to assess equal loading.

### ELISAs for GPNMB and nuclear MITF

Cell or nuclear extracts were prepared from both treated and control HepG2 cells in cell lysis buffer and from ARSB-null and control mouse liver. GPNMB was detected in the whole cell lysate, whereas MITF concentration was determined in the nuclear extract, prepared as above. Nuclear MITF (MyBioSource, Inc., San Diego, CA) and cellular GPNMB (R&D Systems, Minneapolis, MN) were measured in cell and tissue samples using sandwich ELISAs (R&D). Microtiter plates were coated with a capture antibody to the molecule of interest. Samples and standards were added to the wells of the microtiter plate, and the antigen in the lysates was captured by the coated antibody on the plate and detected with biotinylated antibody and HRP-conjugated streptavidin. Hydrogen peroxide / tetramethylbenzidine (TMB) substrate was used to develop the color which was proportional to the bound HRP. The reaction was stopped and the optical density of the color was read at 450 nm in a plate reader (FLUOstar, BMG Labtech, Cary, NC). The concentration in the sample was extrapolated from a standard curve derived using known concentrations.

### Fast-activated cell-based ELISA for phospho-p38 and total p38 MAPK

Fast-activated cell-based ELISA (FACE; In-Cell Western, Active Motif) was used to detect phospho-p38, when phosphorylated at Thr180/Tyr182, and total p38 (Cell Signaling, Danvers, MA), regardless of phosphorylation state, in the combined alpha, beta, gamma, and delta isoforms of p38 MAPK, in the HepG2 cells and in the ARSB-null mice. The ratio of phospho-p38 to total p38 was determined following silencing of ARSB and transfection with different SHP2 vectors.

### Co-immunoprecipitation of SHP2 with chondroitin 4-sulfate

Control or ARSB-silenced HepG2 cell lysates were mixed and incubated with dynabeads (Life Technologies, Carlsbad, CA), which were coated with specific chondroitin 4-sulfate (C4S) antibody (Ly111). C4S and C4S-bound molecules were immunoprecipitated, and immunoprecipitates were subjected to SDS-PAGE and then Western blotting with SHP2 primary antibody and anti-mouse IgG-HRP secondary antibody (R&D). Chemiluminescence of immunoreactive bands was detected and photographed, and band intensity analyzed using Image J software.

### Analysis of GPNMB promoter activity

Human GPNMB promoter construct in a *Renilla reniformis* luciferase reporter gene (Ren SP) vector (LightSwitch Assay, SwitchGear Genomics, Menlo Park, CA) was used to determine differences in GPNMB promoter activation following ARSB silencing by siRNA and exposure to the SHP2 inhibitor PHSP1 (30μM, Sigma-Aldrich), either alone or in combination. The β-actin promoter (GoClone^TM^) construct with *Renilla* luciferase reporter was used as a positive control, and a scrambled sequence (R01) with *Renilla* luciferase reporter was the negative control. These controls showed the effectiveness of transcription and the specificity of the reactions. Transfections were performed when cells were 60–70% confluent, following silencing or treatment for 24 h, using FuGENE HD transfection reagent and the proprietary LightSwitch assay reagent (SwitchGear). After incubation for 24 h, luminescence was measured at 480 nm in a microplate reader (FLUOstar, BMG Labtech) and compared among the different cell preparations.

### Chromatin immunoprecipitation assay for MITF binding to the GPNMB promoter

Binding of MITF to the GPNMB promoter was assessed by chromatin immunoprecipitation (ChIP) assay (ChIP Assay, Active Motif, Carlsbad, CA). For ChIP, IgG control, control silenced, vector control, ARSB-silenced, PHSP1-treated, and ARSB-overexpressed HepG2 cells were fixed with 1% formaldehyde for 10 min at room temperature. This was followed by shearing of chromatin by sonication. The sheared DNA and control IgG were incubated with rabbit polyclonal anti-MITF (MyBioSource) for 1 h. Protein-DNA complexes were precipitated by protein A/G-coupled magnetic beads, and the DNA was purified from the immunoprecipitated complexes by reversal of cross-linking, followed by proteinase K treatment. Then, real-time RT-PCR was performed using Brilliant SYBR Green QRT-PCR Master Mix (Stratagene, La Jolla, CA) and Mx3000 (Stratagene) to amplify the GPNMB promoter. The sequences for the GPNMB promoter were: 5′- CACATCCTGGCACAACATTC-3′ (left) and 5′- CACAGTTACGCACCCTTTCC-3′ (right) and encompassed a putative MITF-binding site (5′-CAGGT-3′) [15–17]. Band intensity was compared among the IgG control, untreated control, control-silenced, vector control, PHSP1-treated, ARSB-silenced, ARSB-overexpressed samples on a 3.0% agarose gel.

### SHP2 activity assay

SHP2 (PTPN11) is a non-receptor tyrosine phosphatase with an SH2 binding domain. SHP2 dephosphorylates p38 MAPK, suppressing p38 MAPK activation [27,28]. SHP2 Duo Set IC activity assay (R&D) was used to measure SHP2 activity in control and treated HepG2 cells and in the liver homogenate of control and ARSB-null mice. In this assay, an anti-SHP2 antibody conjugated to agarose immunoprecipitation (IP) beads was used to pull down both active and inactive SHP2 in the samples. After washing away unbound material, a synthetic phosphopeptide substrate was added to the immunoprecipitates. The substrate was dephosphorylated by active SHP2 in the sample to generate free phosphate and unphosphorylated peptide. The beads were then pelleted by centrifugation and the supernatant was transferred to a microplate. The amount of free phosphate in the supernatant was determined by a sensitive dye-binding assay using malachite green and molybdenic acid. The activity of SHP2 was determined from the phosphate standard curve.

### Confocal imaging of ARSB and GPNMB in HepG2 cells

HepG2 cells were grown in 4-chamber tissue culture slides for 24 hours and stained, as previously [25]. Cells were washed once in 1x PBS containing 1 mM calcium chloride (pH 7.4), and fixed for 1.5 hours with 2% paraformaldehyde. Cells were washed with PBS with 1 mM calcium chloride and 0.08% saponin. Then, cells were blocked with 5% normal horse serum, incubated overnight with rabbit ARSB polyclonal antibody (1:100; Open Biosystems, GE Dharmacon, Lafayette, CO) at 4°C, then washed and stained with anti-rabbit Alexa Fluor® IgG 488 (green, 1:100, Invitrogen, Carlsbad, CA). Preparations were stained with goat GPNMB antibody and with anti-goat Alexa Fluor® IgG 568 (orange-red, Invitrogen)**.** Cells were mounted with DAPI (4’,6-diamidino-2-phenylindole; ProLong gold antifade reagent with DAPI, Invitrogen)-containing mounting media for nuclear staining. Slides were coverslipped and observed using a Zeiss LSM 510 laser scanning confocal microscope equipped with a 63x water-immersion objective. The fluorochromes were scanned sequentially, and the collected images were exported with Zeiss LSM Image Browser software as TIFF files.

### Statistical analysis

Results are the mean ± standard deviation (SD) of at least three independent biological samples with two technical replicates of each assay. Statistical significance was determined by one-way analysis of variance followed by the Tukey-Kramer post-test to correct for multiple comparisons, using InStat3 software (GraphPad, San Diego, CA), unless stated otherwise. A p-value of ≤0.05 was considered statistically significant. In the figures, *** indicates p ≤ 0.001, ** indicates 0.001< p≤0.01, and * indicates 0.01<p≤0.05.

## Results

### Decline in ARSB activity increases GPNMB expression

**Fig 1:**
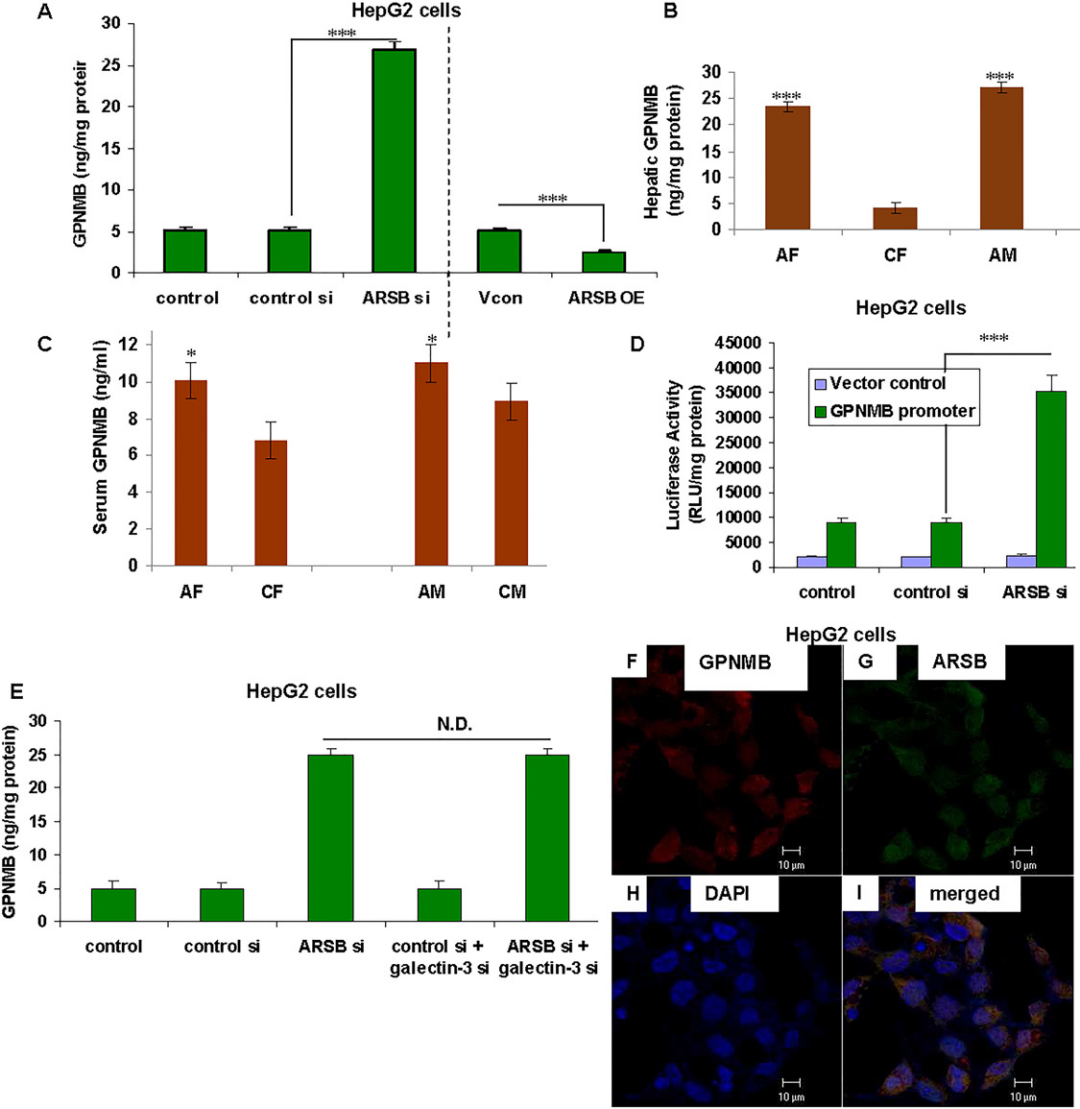
Increase in GPNMB following ARSB decline in hepatic cells and in ARSB-deficient mice is not mediated by galectin-3. **(A)** GPNMB protein was measured by ELISA in the HepG2 cells following ARSB knockdown. The level increased from 5.1 ± 0.4 ng/mg protein to 26.8 ± 1.1 ng/mg protein when ARSB was silenced by siRNA (p<0.001; one-way ANOVA with Tukey-Kramer post-test; n = 6)). Following ARSB overexpression, GPNMB declined to 2.4 ± 0.2 ng/mg protein (p<0.001, n = 6). **(B)** In hepatic tissue from ARSB-null mice, gpnmb mRNA expression increased to more than 5.5 times the baseline in both female and male mice, compared to controls (p<0.001; n = 5 per group). **(C)** GPNMB also increased in the serum of the ARSB-null female and male mice, compared to normal, gender-matched controls (p<0.05, unpaired t-test, two-tailed; n = 5 per group). **(D)** GPNMB promoter activity was detected by luciferase assay, and increased to ~3.5 times the baseline level when ARSB was silenced (p<0.001, n = 6). **(E)** In the HepG2 cells, galectin-3 silencing did not inhibit the increase in GPNMB that followed ARSB silencing (n = 3). **(F,G,H,I)** In the HepG2 cells, confocal images show prominent GPNMB staining (**F**, red) and ARSB staining (**G**, green) diffusely throughout the cytoplasm. Co-localization is apparent in the merged image (**I,** yellow). Nuclei are stained blue by DAPI (**H**). [AF = ARSB-null female; AM = ARSB-null male; ARSB = arylsulfatase B; GPNMB = glycoprotein (transmembrane) NMB; CF = control female; CM = control male; N.D. = no difference; OE = overexpression; si = siRNA; Vcon = vector control]

In a cDNA microarray of hepatic tissue from ARSB-null mice, GPNMB was the only gene that was significantly upregulated [1]. GPNMB expression increased 9.5 fold, compared to wild-type C57BL/6J control, and further studies were initiated to confirm this increase and to investigate the mechanism by which decline in ARSB produced this marked increase. GPNMB protein was measured in control and ARSB-silenced HepG2 cells, and in the liver tissue of control and ARSB-null mice**.** GPNMB increased from 5.1 ± 0.4 ng/mg protein to 26.8 ± 1.1 ng/mg protein following ARSB silencing (p<0.001, one-way ANOVA with Tukey-Kramer post-test), and declined to 2.4 ± 0.2 ng/mg protein when ARSB was overexpressed (p<0.001) (n = 6 per group) (**Fig 1A**). In the hepatic tissue of ARSB-null male and female mice, the GPNMB concentration was 27.1 ± 2.7 and 23.5 ± 2.8 ng/mg protein, respectively. In contrast, in control male and female hepatic tissue, GPNMB concentrations were 4.6 ± 0.4 and 4.2 ± 0.6 ng/mg protein, respectively (total n = 20) (**Fig 1B**). Serum levels were also increased in the ARSB-deficient female and male mice (p = 0.05, unpaired t-test, two-tailed) (**Fig 1C**). These findings confirmed the results of the cDNA microarray, and indicated marked increases in GPNMB expression following ARSB decline.

### Increase in GPNMB expression was not mediated by Galectin-3

GPNMB promoter activity was detected by luciferase assay in the HepG2 cells. Promoter activity increased to 3.92 times the baseline level when ARSB was silenced, (p<0.001; n = 6) (**Fig 1D**), confirming that a transcriptional mechanism was involved. In previous work, transcriptional effects of decline in ARSB and the resulting increase in chondroitin 4-sulfation were mediated by reduced binding of galectin-3 to C4S, leading to increased nuclear galectin-3 [18,19]. Nuclear galectin-3 then acted with AP-1 or with Sp1 to enhance transcriptional events. However, in the HepG2 cells, silencing galectin-3 did not inhibit the increase in GPNMB which followed ARSB knockdown (**Fig 1E**). Hence, galectin-3 was not implicated in the transcriptional mechanism by which decline in ARSB increased GPNMB expression. Confocal images demonstrate the co-localization of ARSB and GPNMB in the HepG2 cells. GPNMB (**Fig 1F**, red). and ARSB (**Fig 1G**, green) co-localize (**Fig 1H**) throughout the cytoplasm and on the cell membrane, yielding a merged yellow image.

Similar effects of ARSB silencing on GPNMB expression were observed in human prostate stromal (3.6 ± 0.3 to 18.3 ± 1.1 ng/mg protein) and epithelial cells (4.8 ± 0.4 to 24.7 ± 1.6 ng/mg protein). Galectin-3 silencing did not inhibit these increases in GPNMB induced by ARSB silencing (data not shown). These findings also indicated that a different transcriptional mechanism was involved in the increased expression of GPNMB that followed decline in ARSB.

### Increases in nuclear MITF and in GPNMB promoter activation follow decline in ARSB

**Fig 2:**
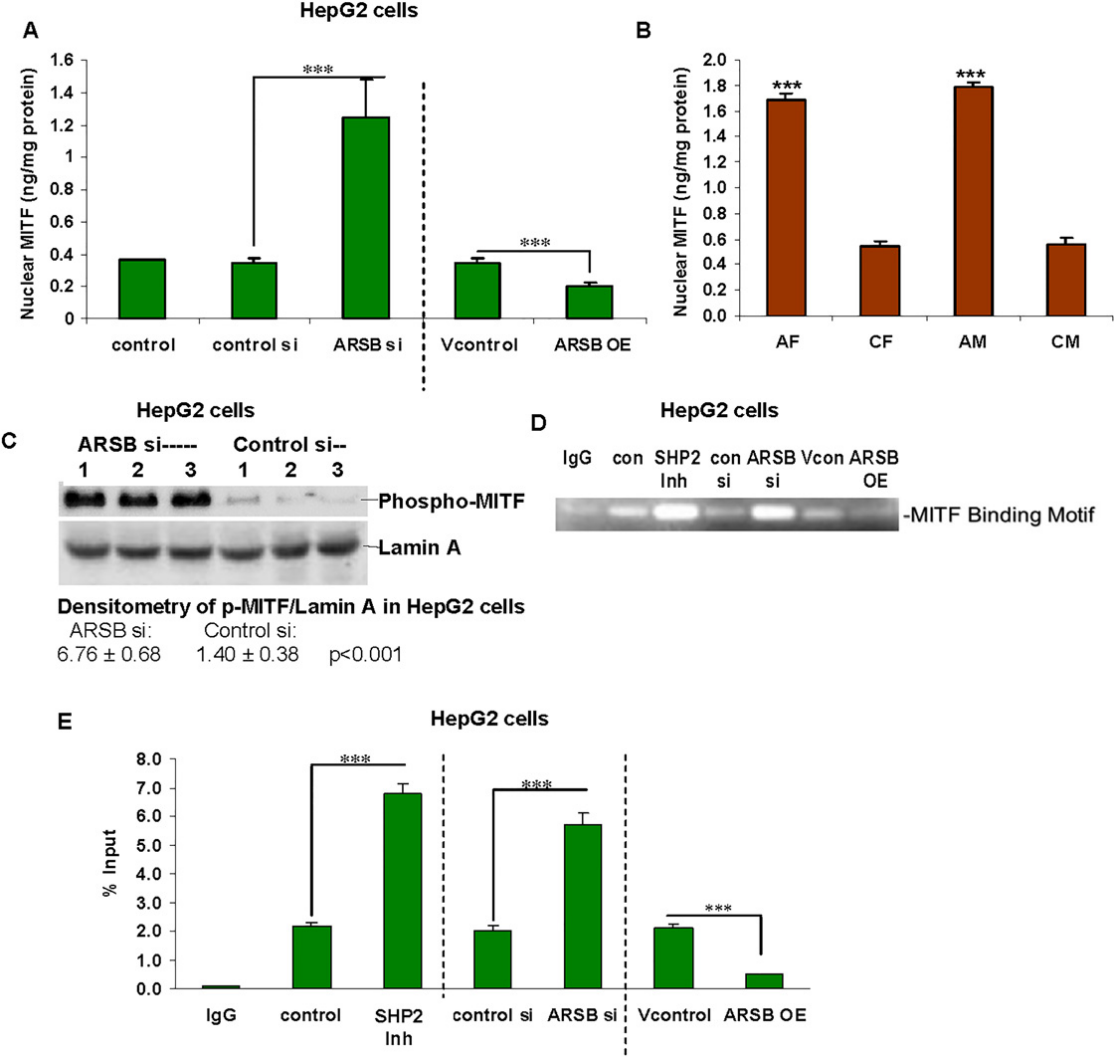
Nuclear MITF and activation of GPNMB promoter follow decline in ARSB. **(A)** Nuclear MITF increased from 0.36 ± 0.001 ng/mg protein to 1.22 ± 0.11 ng/mg protein following ARSB silencing (p<0.001,n = 6). Nuclear MITF declined to 0.20 ± 0.02 ng/mg protein when ARSB was overexpressed (p = 0.003, unpaired t-test, two-tailed; n = 3). **(B)** In the hepatic tissue of the ARSB-null female and male mice, nuclear MITF increased to ~3 times the level in the normal age- and gender-matched C57BL/6J mice (p<0.001, n = 12). **(C)** Western blot shows marked increase in density of the bands for nuclear phospho-MITF in the HepG2 cell following ARSB silencing. The nuclear envelope protein Lamin A was used as control. Densitometry showed that intensity increased to ~4.8 times the control level. **(D)** Chromatin immunoprecipitation assay demonstrated increased binding to the MITF motif in the GPNMB promoter following ARSB silencing and reduced binding following ARSB overexpression in the HepG2 cells (n = 6). Treatment of the cells with the SHP2 inhibitor (PHSP1) also increased the binding to the MITF motif. **(E)** Densitometry of the MITF binding to the GPNMB promoter increased following ARSB knockdown or treatment with the SHP2 inhibitor PHSP1. Following ARSB overexpression, MITF binding declined significantly. [AF = ARSB-null female; AM = ARSB-null male; ARSB = arylsulfatase B; CF = control female; ChIP = chromatin immunoprecipitation; CM = control male; con = control; GPNMB = glycoprotein (transmembrane) NMB; Inh = inhibitor; MITF = microphthalmia-associated transcription factor; N.D. = no difference; OE = overexpression; SHP2 Inh = PHSP1 = SHP2 inhibitor; si = siRNA; Vcon = vector control]

Published reports in other cells indicated that the transcription factor MITF was required for the increase in GPNMB expression [15–17]. This suggested that MITF might be involved in the transcriptional effect of ARSB on GPNMB in the hepatic cells and tissue. Measurement of nuclear phospho(Ser180,Ser73)-MITF in the control and ARSB-silenced HepG2 cells demonstrated an increase in nuclear MITF to 1.22 ± 0.11 ng/mg protein from a control value of 0.36 ± 0.001 ng/mg protein (**Fig 2A**). ARSB overexpression reduced the nuclear MITF to 0.20 ± 0.02 ng/mg. Measurement of nuclear MITF in the liver tissue of control and ARSB-null mice also demonstrated an increase in nuclear MITF to more than 300% the baseline value in both male and female mice (p<0.001) (**Fig 2B**). Western blot confirmed the increase in nuclear p-MITF in the HepG2 cells following ARSB silencing (**Fig 2C**).

The mechanism by which decline in ARSB increased GPNMB was further evaluated in relationship to increase in nuclear MITF. Chromatin immunoprecipitation (ChIP) assay demonstrated increased binding of phospho-MITF to the MITF consensus sequence (5′-CAGGT-3′) in the GPNMB promoter when ARSB was silenced and reduced binding when ARSB was overexpressed (**Fig 2D**). The DNA binding % increased from the control value of 2.3 ± 0.2% to 5.7 ± 0.4% in ARSB-silenced HepG2 cells (**Fig 2E**). Inversely, ARSB overexpression inhibited the binding of MITF to the GPNMB promoter (p<0.001; n = 6). These findings indicated that changes in ARSB regulated the GPNMB promoter activation through effects on MITF. (The ChIP assay was also performed using PHSP1, an SHP2 inhibitor, as detailed below.)

### p38 MAPK inhibitor blocks increases in GPNMB expression and nuclear MITF

**Fig 3:**
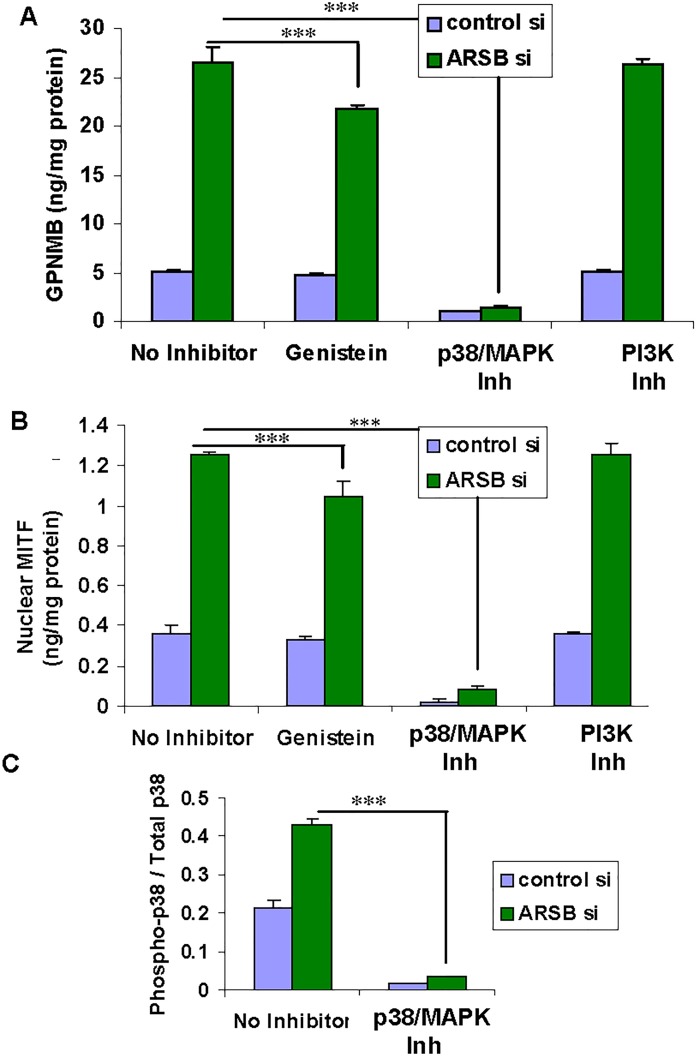
Phospho-p38 inhibitor blocks increases in GPNMB and nuclear MITF. **(A)** When HepG2 cells were treated with the phospho-p38 MAPK inhibitor SB20350, the ARSB siRNA- induced increase in GPNMB declined from 26.4 ± 1.7 ng/mg protein to 1.4 ± 0.2 ng/mg protein (p<0.001, n = 3). Baseline GPNMB declined >80% (from 5.1 ± 0.2 ng/mg protein to 1.0 ± 0.04 ng/mg protein) following exposure to SB20350. Genistein also significantly reduced GPNMB, to 21.7 ± 0.4 ng/mg protein (p<0.001, n = 3), but the PI3K/AKT inhibitor (LY294002) had no effect. **(B)** The increase in nuclear MITF induced by ARSB siRNA was inhibited by SB20350, declining from 1.25 ± 0.02 ng/mg protein to 0.084 ± 0.015 ng/mg protein (p<0.001; n = 3). Genistein also significantly inhibited the ARSB siRNA-induced increase (to 1.04 ± 0.07 ng/mg protein; p<0.001; n = 3), but LY294002 had no effect. **(C)** The phospho-p38 MAPK to total p38 ratio was determined by fast-activated cell-based ELISA. The ratio more than doubled following ARSB silencing (from 0.21 ± 0.02 to 0.43 ± 0.02), and declined significantly following exposure to SB20350 (to 0.015 ± 0.002 with control siRNA and to 0.032 ± 0.003 with ARSB siRNA), demonstrating the effectiveness of the inhibitor. [ARSB = arylsulfatase B; GPNMB = glycoprotein (transmembrane) NMB; MITF = microphthalmia-associated transcription factor; si = siRNA]

The impact of several inhibitors on the expression of GPNMB following ARSB silencing was determined. SB203580, a p38 MAPK inhibitor, very strongly inhibited the expression of GPNMB protein in ARSB-silenced and control cells at a concentration of 10μM x 24 h (p<0.001) (**Fig 3A**). Inhibition of the tyrosine kinase pathway by genistein (10μM x 24 h) also reduced the GPNMB level in the ARSB-silenced cells (p<0.001). The PI3K/AKT inhibitor, LY294002 (50μM x 24h), had no effect on GPNMB expression in either ARSB- silenced or control HepG2 cells. Decline in GPNMB expression was not attributable to an effect of these inhibitors on cell survival, since cell survival was ~95% in both untreated and treated cells. Also, the mRNA expression of lysosomal enzymes, including N-acetylgalactosamine 6-sulfatase, α-galactosidase A, and LFEB, was not reduced following ARSB knockdown, indicating that a generalized effect on lysosomal biogenesis did not occur.

The increase in nuclear MITF that followed ARSB silencing was profoundly inhibited by the phospho-p38 MAPK inhibitor SB203580 and by genistein, but not by the PI3K/AKT inhibitor (**Fig 3B**). Effectiveness of the p38 phosphorylation inhibitor was shown by the marked decline in the phospho-p38 to total p38 ratio following treatment (**Fig 3C**).

### SHP2 activity declines when ARSB is reduced and increases with ARSB overexpression

**Fig 4:**
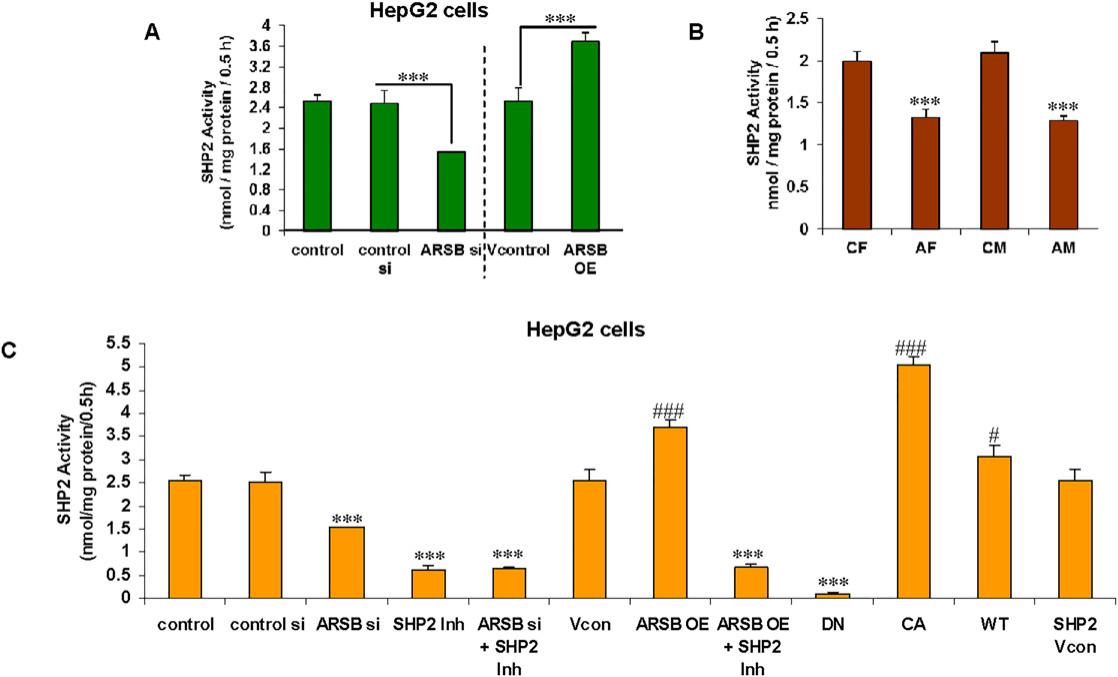
Decline in SHP2 activity follows decline in ARSB in HepG2 cells and in ARSB-null mice. **(A)** The SHP2 activity declined ~36% from (2.49 ± 0.15 nmol/mg protein/0.5 h to 1.59 nmol/mg protein/0.5h) following ARSB silencing (p<0.001; n = 6), and increased to 3.70 ± 0.16 nmol.mg protein/0.5h when ARSB was overexpressed in the HepG2 cells (p<0.001; n = 3). **(B)** SHP2 activity was markedly reduced in the ARSB-null mice (declining from 1.99 ± 0.12 to 1.33 ± 0.10 nmol/mg protein/0.5h, p = 0.001 (F); and 2.09 ± 0.13 to 1.28 ± 0.06 nmol/mg protein/h, p = 0.0006 (M), unpaired t-test, two-tailed; n = 3 per group). **(C)** The impact of different SHP2 DNA constructs on the SHP2 activity was tested. Constitutively active(CA)- and wild-type(WT)-SHP2 DNA increased the SHP2 activity (p<0.001, p<0.05), and dominant negative (DN)-SHP2 DNA inhibited the SHP2 activity (p<0.001). ARSB siRNA, the SHP2 inhibitor PHSP1, and PHSP1 in combination with ARSB siRNA or with ARSB OE reduced the activity (p<0.001; n = 3), in comparison to the controls. SHP2 activity in the CA was ~50 times greater than in the DN (5.04 ± 0.10 nmol/mg protein/0.5h vs. 0.10 ± 0.01 nmol/mg protein/0.5h). [AF = ARSB-null female; AM = ARSB-null male; ARSB = arylsulfatase B; CA = constitutively active; CF = control female; CM = control male; DN = dominant negative; OE = overexpression;si = siRNA; WT = wild-type; * for decrease; # for increase]

Since SHP2 is required for the tyrosine dephosphorylation of p38 MAPK and p38 phosphorylation was implicated in the increases in nuclear MITF and GPNMB, the activity of SHP2 (expressed as nmol / mg protein / 0.5hr) in the ARSB null mice and in the HepG2 cells was evaluated following ARSB silencing and overexpression. In the ARSB-silenced HepG2 cells, SHP2 activity declined significantly (p<0.001), but increased when ARSB was overexpressed (p<0.001) (**Fig 4A**). SHP2 activity was also significantly reduced in the hepatic tissue of the ARSB null mice (male ~40% reduction and female ~34% reduction) (p<0.001; n = 12) (**Fig 4B**).

The effects of different SHP2 DNA constructs were tested in the HepG2 cells using the SHP2 activity assay (**Fig 4C**). As expected, constitutively active (CA) and wild-type (WT) SHP2 DNA increased the SHP2 activity. Dominant negative (DN)-SHP2 DNA construct reduced the activity. The SHP2 chemical inhibitor PHSP1 [35] markedly inhibited the SHP2 activity, and the combination of PHSP1 and ARSB silencing did not further inhibit the SHP2 activity.

### Decline in SHP2 increases phospho-p38 to total p38 ratio, nuclear MITF, GPNMB promoter activation, and GPNMB

**Fig 5:**
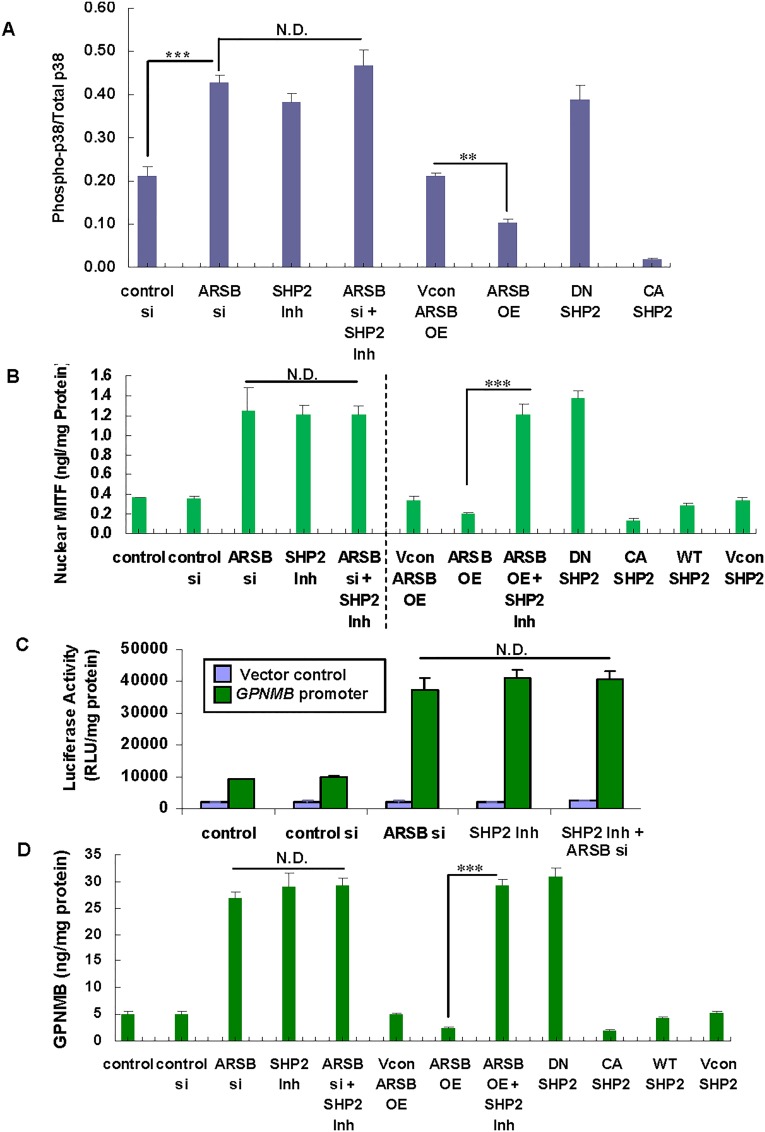
Decline in SHP2 leads to increases in phospho-p38 to total p38 ratio, nuclear MITF, GPNMB promoter activity, and GPNMB in HepG2 cells. **(A)** The ratio of phospho-p38 to total p38 increased to 0.43 ± 0.02 following ARSB siRNA and to 0.38 ± 0.02 following PHSP1 treatment from a control level of 0.21 ± 0.02 (p<0.001; n = 5). DN-SHP2 DNA also increased the ratio (to 0.39 ± 0.03). In contrast, ARSB overexpression (p<0.01) and CA-SHP2 DNA construct (p<0.001) decreased the ratio to 0.10 ± 0.01 and 0.02 ± 0.002, respectively.**(B)** Nuclear MITF increased following ARSB silencing (1.25 ± 0.23 ng/mg protein) and treatment with the DN-SHP2 DNA vector (to 1.37 ± 0.08 ng/mg protein) and the SHP2 inhibitor PHSP1 (1.20 ± 0.10 ng/mg protein) from a control level of 0.36 ± 0.001 ng/mg protein(p<0.001; n = 5), in contrast to the declines following ARSB overexpression or treatment with the CA-SHP2 or WT-SHP2 DNA vectors. PHSP1 treatment of cells in which ARSB was overexpressed reversed the decline in nuclear MITF (p<0.001; n = 5). **(C)** GPNMB promoter activity was increased by PHSP1 to ~4.4 times the control level (p<0.001; n = 5) and by ARSB silencing (to ~4.0 times control), as previously noted (**Fig 2C**). The combination of ARSB siRNA and PHSP1 had no greater effect. **(D)** GPNMB protein was increased by ARSB silencing, PHSP1, and DN SHP2 and reduced by ARSB overexpression, CA SHP2, and WT SHP2. Increase was greatest following CASHP2, increasing to 30.9 ± 1.7 ng/mg protein from control level of 5.13 ± 0.13 ng/mg protein. [ARSB = arylsulfatase B; CA = constitutively active; DN = dominant negative; GPNMB = glycoprotein (transmembrane) NMB; Inh = inhibitor; MITF = microphthalmia-associated transcription factor; OE = overexpression; RLU = relative luciferase units; si = siRNA; Vcon = vector control; WT = wild-type]

The phosphorylation of p38 MAPK was detected by fast-activated cell-based ELISA in treated and control HepG2 cells, following transfection with the SHP2 DNA vectors described above. Increase in the phospho-p38 to total p38 ratio followed treatment with ARSB siRNA, the SHP2 inhibitor PHSP1, or transfection by DN-SHP2 DNA (p<0.001; n = 5). The phospho-p38 to total p38 ratio increased by >100%, 75% and 80%, respectively, due to these treatments (**Fig 5A**). In contrast, ARSB overexpression reduced the ratio of phospho-p38 to total p38 by more than 50% (p<0.01), and transfection with CA-SHP2 DNA vector (p<0.001) decreased the ratio to less than 10% of the control value. PHSP1 treatment of ARSB-silenced HepG2 cells had no additional impact on the ratio.

In addition to its critical role in the regulation of p38 phosphorylation, the effects of SHP2 on phospho-MITF, on the GPNMB promoter activation, and on GPNMB expression were determined. Nuclear MITF increased to 1.25 ± 0.23, 1.20 ± 0.01 and 1.37 ± 0.08 ng/mg protein in ARSB-silenced, PHSP1-treated, and DN-SHP2 transfected cells, respectively, from a control value of 0.36 ± 0.001 ng/mg protein (p<0.001; n = 5) (**Fig 5B**). In contrast, ARSB overexpression and transfection with CA-SHP2 or WT-SHP2 DNA vector decreased the nuclear MITF (range: 0.14 ± 0.01 to 0.28 ± 0.02 ng/mg protein) from the baseline level. PHSP1 treatment of the cells in which ARSB was overexpressed nullified the inhibitory effect of ARSB overexpression and increased the nuclear MITF to 1.21 ± 0.09 ng/mg protein (p<0.01; n = 5).

GPNMB promoter activation was determined by luciferase assay, which demonstrated that ARSB silencing or inhibition of SHP2 by PHSP1 (30μM x 24h) stimulated the GPNMB promoter activity more than 3-fold in comparison to control values (p<0.001; n = 5) (**Fig 5C**). The combination of ARSB silencing and PHSP1 had no greater effect than PHSP1 or ARSB siRNA alone. Chromatin immunoprecipitation (ChIP) assay demonstrated increased binding of phospho-MITF to an MITF consensus sequence (5′-CAGGT-3′) in the GPNMB promoter when ARSB was silenced or following treatment with the SHP2 inhibitor (PHSP1, 30μM x 24 h) (**Fig 2D and 2E**). The DNA binding % increased from the control value of 2.3 ± 0.2% to 5.7 ± 0.4% and 6.8 ± 0.3% in the ARSB-silenced and PHSP1-treated groups, respectively.

Both ARSB silencing and PHSP1 increased the GPNMB protein content (p<0.001; n = 3), and the combination of ARSB siRNA and PHSP1 did not show any additive effect on GPNMB expression. Transfection by DN-SHP2 DNA vector increased the GPNMB content to ~6 times the baseline value. The SHP2 inhibitor, PHSP1, increased the GPNMB level to 29.0 ± 2.7 ng/mg protein, and PHSP1 reversed the inhibitory effect of ARSB overexpression. ARSB overexpression and transfection by CA-SHP2 decreased GPNMB content ~50% in the HepG2 cells (**Fig 5D**).

Overall, these experiments indicated that inhibition of SHP2 increased the phospho-p38 to total p38 ratio, nuclear MITF, GPNMB promoter activation, and total GPNMB. In contrast, WT-SHP2, CA-SHP2, and ARSB overexpression reduced these parameters.

### SHP2 activity is regulated by interaction with C4S

**Fig 6:**
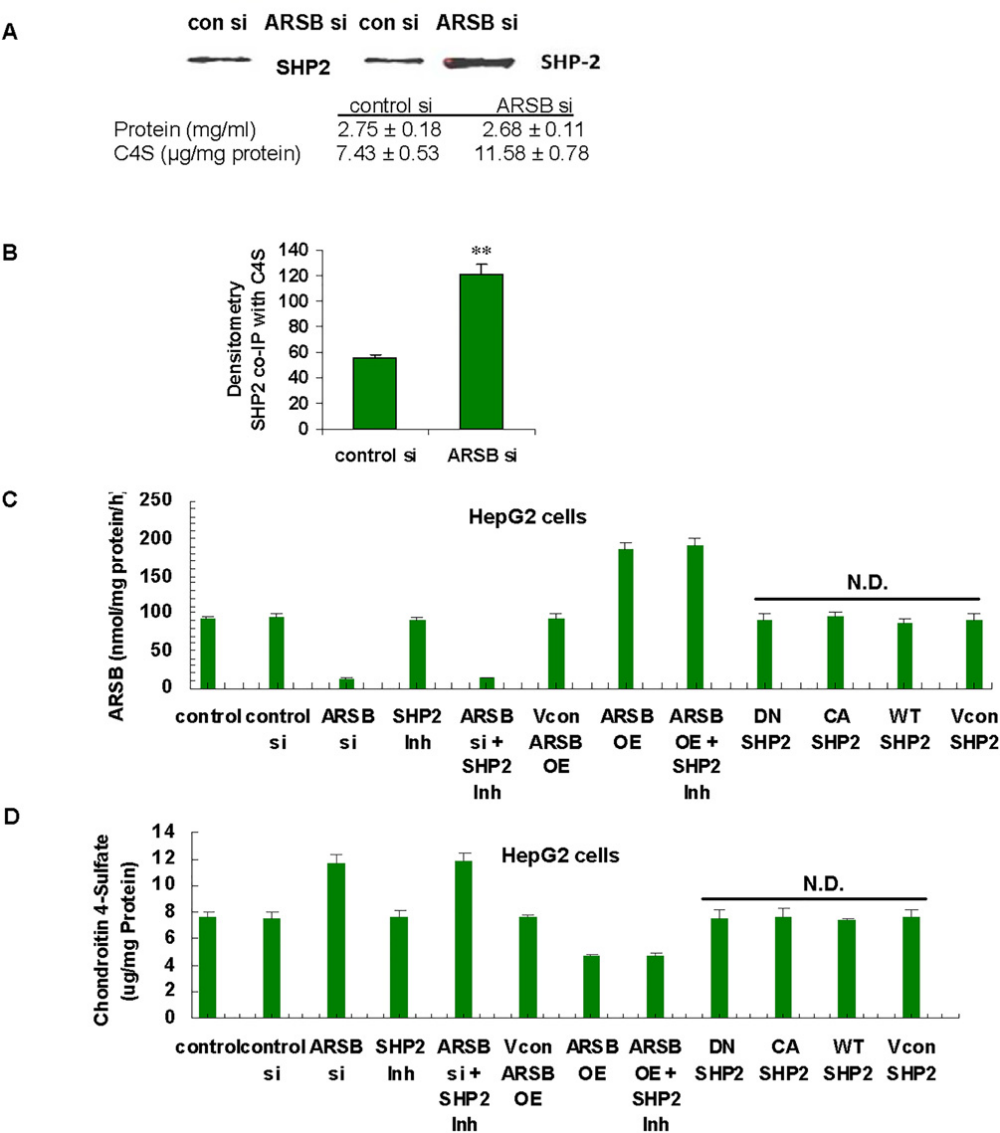
Increase in SHP2 that co-immunoprecipitates with C4S following ARSB knockdown. **(A)** Western blot of SHP2 co-immunoprecipitated with C4S shows increased band density following ARSB silencing compared to control silencing (n = 2). The C4S content increased following ARSB silencing, with similar protein. **(B)** Densitometry showed increased intensity of the immunoprecipitated bands following ARSB silencing (p = 0.007, unpaired t-test, two-tailed; n = 2). **(C)** HepG2 cells were treated with PHSP1, a chemical SHP2 inhibitor and with DN, CA, or WT SHP2 DNA vectors. These exposures had no effect on the ARSB activity. (p>0.05, one-way ANOVA with Tukey-Kramer post-test; n = 3). **(D)** Treatment with PHSP1 or with DN. CA, or WT SHP2 DNA vectors did not modify the chondroitin 4-sulfate in the HepG2 cells. [ARSB = arylsulfatase B; CA = constitutively active; con si = control siRNA; DN = dominant negative; Inh = inhibitor; N.D. = no difference; OE = overexpression; si = siRNA; Vcon = vector control; WT = wild-type]

The mechanism by which ARSB and C4S regulated the effects of SHP2 on downstream phosphorylations was addressed. Based on previous findings that C4S bound vital molecules more or less tightly depending on the chondroitin 4-sulfation, ARSB-silenced and control HepG2 cell extracts were immunoprecipitated with a specific antibody to C4S, and the immunoprecipitates were subjected to Western blot with specific antibody to SHP2. When ARSB activity was reduced and chondroitin 4-sulfation increased, the co-immunoprecipitation of SHP2 with C4S increased (**Fig 6A**). Densitometry demonstrated the significant increase in the SHP2 that co-immunoprecipitated with C4S (p = 0.007, unpaired t-test, two-tailed) following ARSB silencing (**Fig 6B**). Hence, interaction of SHP2 with more sulfated C4S reduced the SHP2 activity, leading to decline in GPNMB expression through effects on phospho-p38 and phospho-MITF.

No direct effect of SHP2 DNA constructs on ARSB activity (**Fig 6C**) or chondroitin 4-sulfate content was detected (**Fig 6D**). ARSB activity was measured in HepG2 cells after ARSB silencing, ARSB overexpression, the SHP2 inhibitor PHSP1, and transfections by DN-SHP2;HP2 CA-SHP2; WT-SHP2, or empty vector. ARSB silencing reduced the ARSB activity from 98.8 ± 4.4 nmol/mg protein/h to 12.8 ± 14.4 nmol/mg protein/h. In contrast, ARSB overexpression increased activity to 185.8 ± 9.6 nmol/mg protein/h. Neither PHSP1 nor DN-SHP2, CA-SHP2 or WT-SHP2 vectors had any significant effect on ARSB activity (**Fig 6C**).

ARSB activity was also measured in control and ARSB-null mouse hepatic tissue. ARSB activity was <3% in the liver of the ARSB-null male and female mice, in comparison to control (p<0.001). SHP2 inhibition or overexpression had no significant effect on C4S content. Hepatic tissue of ARSB-null mice had ~55% and ~56% more C4S in female and male mice than in the gender-matched controls. Measurement of C4S content in treated HepG2 cells demonstrated a ~50% increase following ARSB silencing and ~38% reduction following ARSB overexpression.

**Fig 7:**
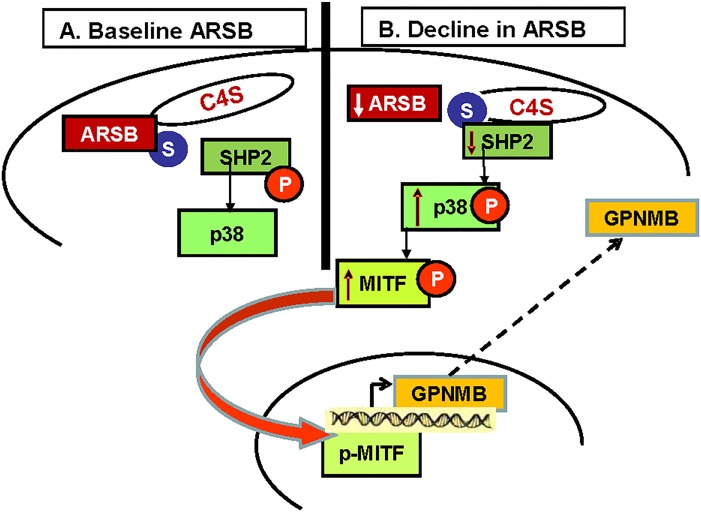
Schematic illustration of signaling pathway from ↓ARSB → ↑chondroitin 4-sulfate → ↑SHP2 bound → ↓SHP2 activity → ↑phospho-p38 → ↑MITF → ↑GPNMB. **(A)** When ARSB activity is normal, the 4-sulfate group at the non-reducing end of the C4S chain is removed from C4S and the binding of SHP2 to C4S is not increased. Hence, SHP2 can remove tyrosine phosphate from p38. **(B)** When ARSB is reduced and chondroitin 4-sulfation is increased, SHP2 binding to C4S is increased and SHP2 activity is reduced. There is inhibition of removal of tyrosine phosphate from p38, leading to increased phosphorylation of p38 and of MITF, leading to activation of the GPNMB promoter. [ARSB = arylsulfatase B; C4S = chondroitin 4-sulfate; GPNMB = glycoprotein (transmembrane) NMB; MITF = microphthalmia-associated transcription factor; P = phosphate; S = sulfate]

Study findings are summarized schematically in **Fig 7**and indicate that ARSB-induced changes in chondroitin 4-sulfation inhibit the SHP2-mediated dephosphorylation of phospho-p38 MAPK, leading to activation of MITF and increased expression of GPNMB.

## Discussion

In this study, we identified the molecular events that increased GPNMB expression in hepatic cells following decline in ARSB, and identified a critical interaction between SHP2 and chondroitin 4-sulfate that regulated the downstream phosphorylation of p38 MAPK and the subsequent activation of the transcription factor MITF. The experiments demonstrated an unanticipated association between chondroitin 4-sulfation and SHP2, and present a second mechanism whereby chondroitin 4-sulfation and ARSB can regulate transcriptional events. Previously, reduced binding of galectin-3 to more highly sulfated chondroitin 4-sulfate was shown to increase expression of versican in human prostate cells and Wnt9A in human colonic epithelial cells [18–20]. Current findings show a mechanism whereby increased binding of SHP2 to more highly sulfated chondroitin 4-sulfate leads to transcriptional effects. Importantly, the findings show a critical interaction between sulfatase and phosphatase activity, with the potential for sulfatase impact on a broad range of cellular effects, since changes in SHP2 action can lead to profound changes in cellular signaling, proliferation, and differentiation [26–28,37].

SHP2 is a non-receptor phosphatase that removes phosphate groups from multiple SH2 tyrosine phosphorylation domains, affecting a wide range of vital cell signaling events. SHP2 has been identified as a tumor suppressor in hepatocellular malignancies, and the inhibition of SHP2 when ARSB is reduced is consistent with the role of ARSB as a tumor suppressor [21–25,39]. The implications of inhibition of phosphate removal by increased binding of SHP2 with C4S are wide-ranging, and include not only the impact on the tyrosine phosphorylations that are directly removed by SHP2. Threonine and serine phosphorylations, such as those of p38 and MITF, are also affected, due to the dual-phosphorylation motif of Thr-Gly-Tyr of residues 180–182 of p38 [27–31].

The proposed mechanism for the promoter activation of GPNMB by phospho-MITF following ARSB silencing is attributable to the increase in chondroitin 4-sulfation following decline in ARSB. ARSB deficiency, either inborn or acquired, thus, can exert a profound effect outside of its direct chemical action to remove sulfate groups from N-acetylgalactosamine 4-sulfate groups of chondroitin 4-sulfate or dermatan sulfate. The location of ARSB on the cell membrane as well as intracellularly, enables interaction with C4S on the cell surface or in the extracellular matrix, in addition to cytoplasmic C4S. This proximity gives ARSB the potential to significantly modulate the interactions between C4S and signaling molecules in the ECM or with SHP2 in the cytoplasm and to impact on transcriptional events. ARSB on the endothelial cell surface [34] may be involved in direct interactions with circulating sulfated GAGs, potentially leading to systemic effects.

In contrast to the impact on transcription mediated by reduced binding of galectin-3 to more highly sulfated chondroitin 4-sulfate, the effect on GPNMB transcription is mediated by the increased binding of SHP2 to more highly sulfated chondroitin 4-sulfate, leading to phosphorylation and activation of the transcription factor MITF and integration of critical sulfatase- and phosphatase-mediated signaling mechanisms. Other important proteins have also been shown to bind more or less tightly to more or less sulfated C4S. Our previous observations include: increased binding of Interleukin-8, high molecular weight kininogen, and bone morphogenetic protein-4 to more highly sulfated C4S, and reduced binding of galectin-3 to more highly sulfated C4S [18–20,32,33,40,41]. Binding of growth factors and other molecules to GAGs, including heparin and hyaluronan, are well-recognized as critical events in cell signaling [42–47].

Arylsulfatase B activity requires post-translational modification by the formylglycine modifying enzyme and oxygen [20,48,49]. Exogenous ions, including chloride, bromide, and phosphate, can interfere with the activation of ARSB [33,50,51], consistent with sensitive responses to ambient conditions. Tight regulation of chondroitin 4-sulfation by small changes in ARSB activity due to effects of oxygen or to high chloride may lead to rapid, small changes in binding of vital signaling molecules. These dynamic changes may regulate cell-cell and cell-matrix signaling interactions, such as those of IL-8 and BMP4, and transcriptional events, such as those mediated by galectin-3 and SHP2. Processes of cell differentiation and cell proliferation may be driven by small changes in the environment, mediated through ARSB-induced changes in chondroitin 4-sulfation affecting the BMP4-Wnt axis [19,41]. Acquired ARSB deficiency, in response to environmental cues or due to subtle changes in the extracellular milieu, may lead to sustained alterations in critical cellular processes

The specific mechanism by which decline in ARSB and the concomitant increase in chondroitin 4-sulfation inhibits the tyrosine phosphatase function of SHP2 is possibly due to binding of positively charged residues of SHP2 with the anionic sulfate group of C4S. Shen *et al* proposed that the transmembrane tyrosine phosphatase PTPσ in astrocytes acts as a membrane receptor for chondroitin sulfate proteoglycans, which participate in formation of the neuronal scar and impede neuronal regeneration following injury [52]. A group of four lysine residues in the Ig-like domain of PTPσ interacted with anionic chondroitin sulfate proteoglycans (CSPGs), such as neurocan. Similar interaction between basic residues of SHP2 and anionic sulfate groups of C4S may lead to enhanced binding between SHP2 and intracellular C4S when ARSB activity is reduced.

Specific extracellular matrix proteoglycans, such as versican, aggrecan, biglycan, and decorin, which are increased in rat hepatocellular carcinomas [53], may affect binding with the C4S attachments on the proteoglycan. Further identification of the precise SHP2 residues that interact with C4S is needed. This information will help to clarify how the regulation of intracellular phosphorylation signaling may be regulated by sulfatase action/inaction on chondroitin 4-sulfation. Other GAG-mediated signaling events may also be regulated by sulfatases, as with activation of abnormal receptor tyrosine kinase signaling by the enzyme SULF2, a heparan sulfate endosulfatase [54].

An issue raised by the study findings is how the increase in GPNMB might compensate for deficiency of ARSB in the ARSB-null mice. GPNMB has an arginylglycylaspartic acid (RGD) domain and interacts with β-1 integrin, thereby contributing to cell-matrix interactions [55]. GPNMB interaction with integrins appears to be a critical feature that enables invasiveness and malignant progression. Also, GPNMB has three arginine-arginine groups, and these positively charged residues may interact with the sulfate groups of C4S, or other negatively charged residues.

Several recent studies have identified high GPNMB expression in malignancies, including in aggressive melanoma, glioblastoma multiforme, hepatocellular carcinoma, and triple negative breast cancers [2–11]. Further characterization of the cell-matrix and intracellular functions of GPNMB, and tissue specific effects, are required to better understand the structural and functional effects of GPNMB. Also, the interactions between ARSB and the MITF-family of transcription factors (TFs), including MITF, TFEB, TFEC, and TFE3, may extend beyond the increased expression of GPNMB. This family of basic helix-loop-helix leucine zipper transcription factors is involved in diverse biological processes, including lysosomal biogenesis, nutrient sensing, and energy metabolism, as well as effects in carcinogenesis, including in melanoma, papillary renal cell carcinoma, and alveolar soft part sarcoma [56–60]. Some of the observed effects of decline in ARSB, increase in chondroitin 4-sulfation, and increase in GPNMB may be attributable to effects on MITF or other MITF-family TFs. Interactions between ARSB and MITF detailed in this report may represent one aspect of a more complex relationship affecting cell survival, and the reported impact of MITF on lysosomal biogenesis may help to restore ARSB function and provide feedback to increase ARSB and inhibit MITF activation [59,60]. Increase in ARSB activity should be considered as a potential target for treatment of malignancies in which increased expression of GPNMB occurs due to increased activation of MITF.
